# Supplementary figures and images for: Agroecosystem edge effects on vegetation, soil properties, and the soil microbial community in the Canadian prairie
Source: PLoS One. 2023 Apr 6;18(4):e0283832. doi: 10.1371/journal.pone.0283832 (PMC10079068; doi:10.1371/journal.pone.0283832)

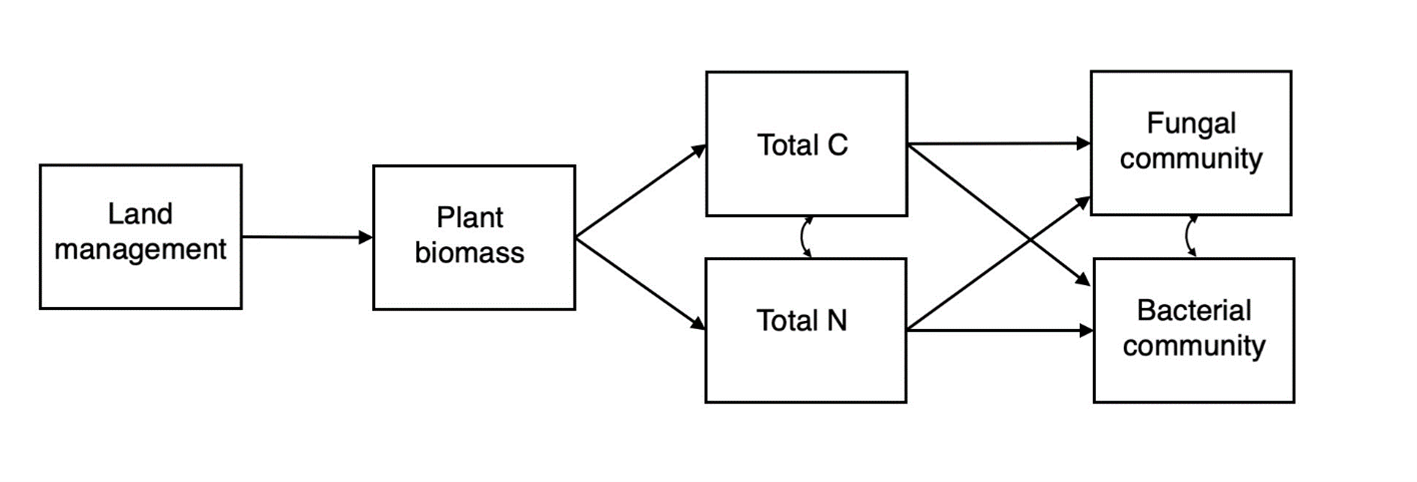

Supplement: S1 Fig — A priori model used for structural equation modelling. Direct relationships are represented by straight arrows and curved arrows represent unexplained covariate relationships. The first and second axes from non-metric multidimensional scaling analyses was used to represent the fungal and bacterial communities. (TIF) [file pone.0283832.s001.tif]

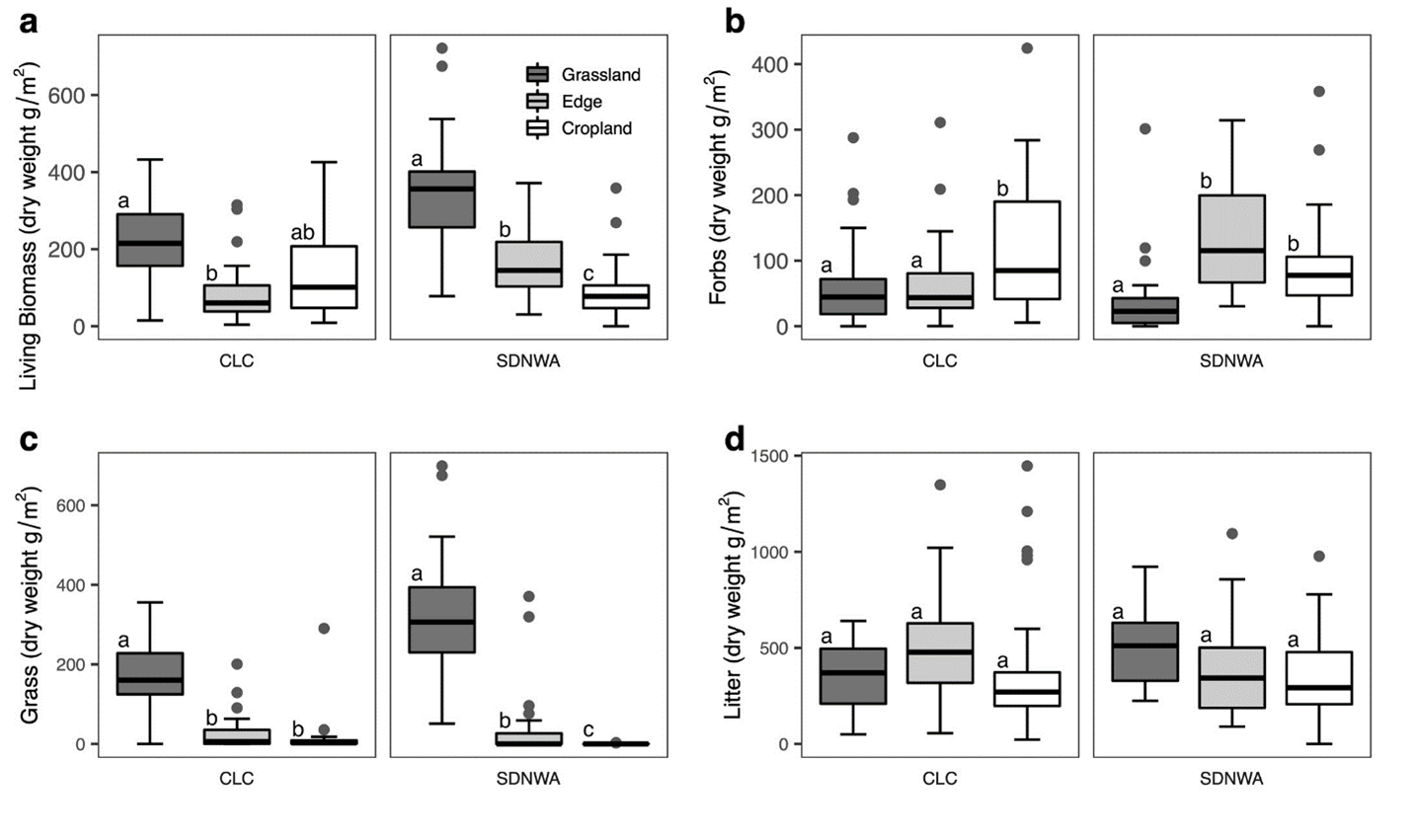

Supplement: S2 Fig — Aboveground vegetation biomass (dry weight g/m2) across edge locations (perennial grassland (dark grey), edge (light grey), and cropland (white) at the Conservation Learning Centre (CLC) and St. Denis National Wildlife Area (SDNWA). Boxes encompass 25–75% quantiles of the data, while whiskers encompass 5–95%. The median is indicated by the black horizontal line, and outliers are shown as dots. Different letters indicate a significant difference (p-value < 0.05) between edge locations determined by Tukey-HSD post-hoc tests on linear mixed models. (TIF) [file pone.0283832.s002.tif]

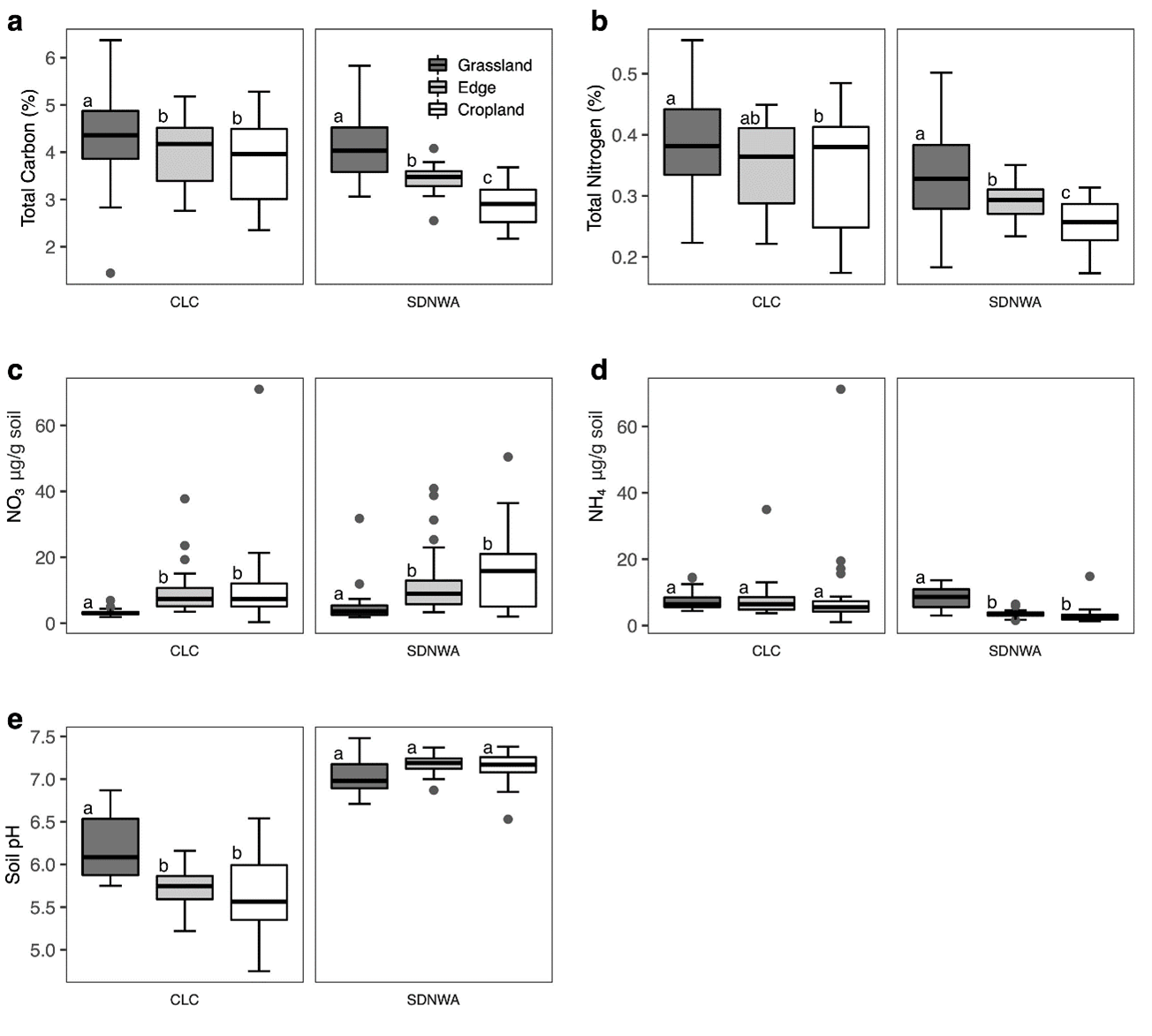

Supplement: S3 Fig — Soil properties across edge locations (perennial grassland (dark grey), edge (light grey), and cropland (white) at the Conservation Learning Centre (CLC) and St. Denis National Wildlife Area (SDNWA). Different letters indicate a significant difference (p-value < 0.05) between edge locations determined by Tukey-HSD post-hoc tests on linear mixed models. (TIF) [file pone.0283832.s003.tif]
