# Supplementary material for: Agroecosystem edge effects on vegetation, soil properties, and the soil microbial community in the Canadian prairie
Source: PLoS One. 2023 Apr 6;18(4):e0283832. doi: 10.1371/journal.pone.0283832 (PMC10079068; doi:10.1371/journal.pone.0283832)
Supplement: S1 Table — Indicator species are also listed with edge + grassland and edge + cropland. Edge + Grassland is the combination of edge and grassland points on the transect, while Edge + Cropland is the combination of edge and cropland points on the transect. (DOCX) [file pone.0283832.s004.docx]

| **Edge Location** | **Plant Species (CLC)** | **Plant Species (SDNWA)** |
| --- | --- | --- |
| Perennial Grassland | Meadow brome (*Bromus bieberstenii* Roem. & Schult.)  Alfalfa (*Medicago sativa* L*.*)  Smooth brome (*Bromus inermis* Lyess)  Kentucky bluegrass (*Poa pratensis* L.)  American vetch (*Vicia americana* Muhl. ex Willd.)  Rocky mountain fescue (*Festuca saximontana* Rydb.)  Showy aster (*Eurybia conspicua* (Lindl.) G.L.Nesom) | Smooth brome (*Bromus inermis* Lyess)  Alfalfa (*Medicago sativa* L*.*)  Dandelion (*Taraxacum officinale* F.H. Wigg*.*)  Kentucky bluegrass (*Poa pratensis* L.)  Slender wheatgrass (*Elymus trachycaulus* (Link) Gould ex Shinners) |
| Edge | Cleaver’s (*Galium aparine* L.)  Hemp nettle (*Galeopsis tetrahit* L.)  unknown grass | Campion (*Silene latifolia* Poir.)  Hemp nettle (*Galeopsis tetrahit* L.)  Hawk’s beard (*Crepis tectorum* L.)  Cleaver’s (*Galium aparine* L.)  Rush skeleton weed (*Chondrilla juncea* L.) |
| Cropland | Canola (*Brassica napus* L.) | Flax (*Linum usitatissimum* L.)  Hoary cress (*Lepidium draba* L.) |
| Edge + Grassland | Dandelion (*Taraxacum officinale* F.H. Wigg*.*)  Canada thistle (*Cirsium arvense* (L.) Scop.)  Perennial sow thistle (*Sonchus arvensis* L.)  Canada goldenrod (*Solidago canadensis* L.) | Canada thistle (*Cirsium arvense* (L.) Scop.)  Perennial sow thistle (*Sonchus arvensis* L.)  Quackgrass (*Elymus repens* (L.) Gould) |
| Edge + Cropland | Canola (*Brassica napus* L.)  unknown grass  Bindweed (*Polygonum convolvulus* L.) | Flax (*Linum usitatissimum* L.)  Flixweed (*Descurainia sophia* (L.) Webb ex Prantl)  Wormseed mustard (*Erysimum cheiranthoides* L.) |
