# Supplementary material for: Agroecosystem edge effects on vegetation, soil properties, and the soil microbial community in the Canadian prairie
Source: PLoS One. 2023 Apr 6;18(4):e0283832. doi: 10.1371/journal.pone.0283832 (PMC10079068; doi:10.1371/journal.pone.0283832)
Supplement: S2 Table — Significant p-values are bolded and log transformed data are denoted by †. (DOCX) [file pone.0283832.s005.docx]

| **Factor** | **Biomass Type** | | | | | | | |
| --- | --- | --- | --- | --- | --- | --- | --- | --- |
|  | df | Living Biomass^†^ | | Grass^†^ | | Forbs^†^ | | Litter^†^ |
| Edge Location | 2 | 28  (**<0.001**) | | 200  (**<0.001**) | | 19  (**<0.001**) | | 4.1  (**0.02**) |
| Site | 1 | 3.03  (0.11) | | 0.50  (0.56) | | 0.28  (0.61) | | 0.04  (0.80) |
| Edge Location x Site | 2 | 8.7  (**<0.001**) | | 12  (**<0.001**) | | 11  (**<0.001**) | | 3.7  (**0.02**) |
|  | **Soil Properties** | | | | | | | |
|  | df | Total C | Total N | | NH_4_^†^ | | NO_3_^†^ | pH |
| Edge Location | 2 | 46  (**<0.001**) | 26  (**<0.001**) | | 25  (**<0.001**) | | 36  (**<0.001**) | 11  (**<0.001**) |
| Site | 1 | 0.95  (0.24) | 1.0  (0.23) | | 11  (**0.03**) | | 5.9  (0.14) | 300  (**<0.001**) |
| Edge Location x Site | 2 | 5.8  (**0.003**) | 2.4  (0.09) | | 17  (**<0.001**) | | 0.36  (0.70) | 31  (**<0.001**) |
