# Supplementary material for: Agroecosystem edge effects on vegetation, soil properties, and the soil microbial community in the Canadian prairie
Source: PLoS One. 2023 Apr 6;18(4):e0283832. doi: 10.1371/journal.pone.0283832 (PMC10079068; doi:10.1371/journal.pone.0283832)
Supplement: S3 Table — Denis National Wildlife Area. (DOCX) [file pone.0283832.s006.docx]

| ***Bacteria*** | |  | | | | | | |
| --- | --- | --- | --- | --- | --- | --- | --- | --- |
| **SDNWA** | |  | | | | | | |
|  | df | | Sum of Sqs | Mean Sqs | F Value | r^2^ |  | *p* |
| Group | 2 | | 2.361 | 1.18052 | 2.4771 | 0.05508 |  | 0.001 |
| Residuals | 85 | | 40.508 | 0.47657 |  | 0.94492 |  |  |
| Total | 87 | | 42.869 |  | | 1 |  |  |
| **CLC** | |  | | | | | | |
|  | df | | Sum of Sqs | Mean Sqs | F Value | r^2^ |  | *p* |
| Group | 2 | | 2.155 | 1.07748 | 2.3367 | 0.05212 |  | 0.001 |
| Residuals | 85 | | 39.195 | 0.46112 |  | 0.94788 |  |  |
| Total | 57 | | 41.35 |  | | 1 |  |  |
| ***Fungi*** |  | |  |  | |  |  |  |
| **SDNWA** |  | |  |  | |  |  |  |
|  | df | | Sum of Sqs | Mean Sqs | F Value | r^2^ | *p* | |
| Group | 2 | | 4.509 | 2.25446 | 3.298 | 0.071 | 0.001 | |
| Residuals | 86 | | 58.789 | 0.68359 |  | 0.92877 |  | |
| Total | 88 | | 63.298 |  |  | 1 |  | |
| **CLC** |  | |  |  |  |  |  | |
|  | df | | Sum of Sqs | Mean Sqs | F Value | r^2^ | *p* | |
| Group | 2 | | 3.086 | 1.54277 | 2.2494 | 0.05027 | 0.001 | |
| Residuals | 85 | | 58.299 | 0.68587 |  | 0.94973 |  | |
| Total | 87 | | 61.384 |  |  | 1 |  | |
