# Supplementary material for: Agroecosystem edge effects on vegetation, soil properties, and the soil microbial community in the Canadian prairie
Source: PLoS One. 2023 Apr 6;18(4):e0283832. doi: 10.1371/journal.pone.0283832 (PMC10079068; doi:10.1371/journal.pone.0283832)
Supplement: S4 Table — (DOCX) [file pone.0283832.s007.docx]

| **Regressions** | **Estimate** | **Standard error** | **z-value** | **p-value** | **Standardized coefficient** |
| --- | --- | --- | --- | --- | --- |
| ***Perennial grassland as land management reference*** | | | | | |
| Plant biomass ~ |  |  |  |  |  |
| Cropland | −1.111 | 0.155 | −7.155 | <0.001 | −0.541 |
| Edge | −0.938 | 0.153 | −6.120 | <0.001 | −0.462 |
| Total carbon ~ |  |  |  |  |  |
| Cropland | −0.771 | 0.159 | −4.841 | <0.001 | −0.432 |
| Edge  Plant biomass | −0.462  0.103 | 0.152  0.068 | −3.031  1.508 | 0.002  0.132 | −0.262  0.119 |
| Total nitrogen ~ |  |  |  |  |  |
| Cropland | −0.051 | 0.016 | −3.164 | 0.002 | −0.299 |
| Edge | −0.033 | 0.015 | −2.147 | 0.032 | −0.197 |
| Plant biomass | 0.007 | 0.007 | 1.078 | 0.281 | 0.090 |
| Fungal community ~ |  |  |  |  |  |
| Total carbon  Total nitrogen | −0.059  0.440 | 0.031  0.309 | −1.903  1.425 | 0.057  0.154 | −0.294  0.208 |
| Cropland | 0.211 | 0.029 | 7.382 | <0.001 | 0.589 |
| Edge | 0.145 | 0.026 | 5.559 | <0.001 | 0.411 |
| Bacterial community~  Total carbon  Total nitrogen  Cropland  Edge | 0.006  0.272  −0.062  −0.069 | 0.032  0.316  0.029  0.027 | 0.203  0.860  −2.105  −2.582 | 0.839  0.390  0.035  0.010 | 0.037  0.149  −0.199  −0.226 |
| ***Edge as land management reference*** | | | | | |
| Plant biomass ~ |  |  |  |  |  |
| Cropland | −0.173 | 0.155 | −1.120 | 0.263 | −0.084 |
| Perennial | 0.938 | 0.153 | 6.120 | <0.001 | 0.460 |
| Total carbon ~ |  |  |  |  |  |
| Cropland | −0.309 | 0.140 | −2.212 | 0.027 | −0.173 |
| Perennial  Plant biomass | 0.462  0.103 | 0.152  0.068 | 3.031  1.508 | 0.002  0.132 | −0.261  0.119 |
| Total nitrogen ~ |  |  |  |  |  |
| Cropland | −0.018 | 0.014 | −1.266 | 0.206 | −0.105 |
| Perennial | 0.033 | 0.015 | 2.147 | 0.032 | 0.96 |
| Plant biomass | 0.007 | 0.007 | 1.078 | 0.281 | 0.090 |
| Fungal community ~ |  |  |  |  |  |
| Total carbon  Total nitrogen | −0.059  0.440 | 0.031  0.309 | −1.903  1.425 | 0.057  0.154 | −0.294  0.208 |
| Cropland | 0.066 | 0.026 | 2.568 | 0.010 | 0.184 |
| Perennial | −0.145 | 0.026 | −5.559 | <0.001 | −0.409 |
| Bacterial community~  Total carbon  Total nitrogen  Cropland  Perennial | 0.006  0.272  0.007  0.069 | 0.032  0.316  0.026  0.027 | 0.203  0.860  0.284  2.582 | 0.839  0.390  0.776  0.010 | 0.037  0.149  0.024  0.225 |
